# Measurement of gantry rotation time in modern ct

**DOI:** 10.1120/jacmp.v15i1.4517

**Published:** 2014-01-06

**Authors:** Atsushi Fukuda, Pei‐Jan P. Lin, Kosuke Matsubara, Tosiaki Miyati

**Affiliations:** ^1^ Department of Radiology Shiga Medical Center for Children Moriyama Shiga Japan; ^2^ Division of Health Sciences Graduate School of Medical Sciences, Kanazawa University Kanazawa Ishikawa Japan; ^3^ Department of Radiology Beth Israel Deaconess Medical Center and Harvard Medical School Boston MA USA

**Keywords:** gantry rotation time, solid‐state detector, CT, temporal resolution

## Abstract

The purpose of this study was to develop and evaluate a noninvasive method to assess rotation time in modern commercial computed tomography (CT) systems. The rotation time was measured at a selected nominal rotation time (400 ms) utilizing two types of solid‐state detectors: the RTI's CT Dose Profiler (CTDP) and Unfors’ Xi (Xi) probes. Either CTDP or Xi was positioned on the inner cover of the gantry and a sheet of lead (1 mm thick) placed on top of the detector. Since a pair of two successive peaks is used to determine the gantry rotation time, by necessity the helical scan must be employed. Upon completion of the data acquisition, these peak times were determined with the dedicated software to obtain rotation time. The average rotation time obtained with CTDP and Xi operated under the dedicated software was found to be 400.6 and 400.5 ms, respectively. The detector for this measurement need not be specifically designed for CT dosimetry. The measurements of CT scanner rotation time can be accomplished with a radiation probe designed for the CT application or a conventional radiation probe designed for radiography and fluoroscopy applications. It is also noteworthy to point out that the measurement results are in good agreement between the two radiation detector systems. Finally, clinical medical physicists should be aware of the accuracy and precision of gantry rotation time, and take into consideration for QA where and when applicable.

PACS number: 87.57.Q‐

## I. INTRODUCTION

With the advent of slip ring technology, helical scan computed tomography (CT) was introduced in 1988.^1^, ^2^ The slip ring technology enabled continuous rotation of CT gantry and, initially, had a gantry rotation time of one second. Ten years after the introduction of single‐row detector CT (SDCT), multirow detector CT scanner (MDCT) was ushered in by several manufacturers.^(7)^ In order to take full advantage of the slip ring technology, further development of existing X‐ray components was inevitable. Among such developments are increased heat capacity of the X‐ray tube and increased speed of gantry rotation, which resulted in the capability to scan a longer anatomic coverage in the same or shorter time. The quest for an ever shorter scan time did not stop at the achievement of one second per rotation scan time.

Temporal resolution is one of the crucial factors in coronary CT angiography,^(4)^ left ventricular,^(5)^ and pediatric CT examinations.^(6)^ Faster imaging time has been an important factor in reducing both motion artifact and need to use sedation or general anesthesia for pediatric patients.

While the gantry rotation time is an important scan parameter of any CT scanner, the accuracy and precision are not necessarily known to medical physicists who have responsibility on quality assurance (QA) of CT scanners. The American Association of Physicists in Medicine (AAPM) published at least two reports on QA of CT scanners, Report 39^(7)^ and Report 83,^(8)^ that dealt with the specification and test procedures. Both reports included evaluation of performance of electromechanical components (e.g., alignment of table to gantry, table motion, collimation, radiation dose profile, and the X‐ray generator kVp accuracy). While the gantry rotation time was not explicitly included in these reports, verification of gantry rotation time is essential to the proper assessment of a modern CT system. However, to the best of our knowledge, there have been no published studies measuring the gantry rotation time.

Solid‐state detector systems were recently developed by several manufactures. They can be employed to measure radiation dose rate in interventional fluoroscopic system^(9)^ and CT,^(10)^ and to record the dose rate profile with high temporal resolution of 1.0 ms or less. Therefore, it can be hypothesized that the latest commercially available solid‐state detector system allows for measurement of gantry rotation time. The aim of this study was to provide a simple noninvasive approach to assess gantry rotation time in a modern commercial CT system.

## II. MATERIALS AND METHODS

### A. CT scanner and two solid‐state detector systems

The MDCT scanner employed for this investigation was equipped with 64 rows of detector array with microdetector width of 0.625 mm. The scan parameters employed were: tube potential of 120 kVp, tube current 100 mA, total collimation width 40.0 mm (0.625 mm×64 rows) and nominal gantry rotation time 400 ms. The investigators had two solid‐state detector systems on hand for the measurement of gantry rotation time, and the specifications of these detectors are shown in Table 1.

The first system is the CT dose profiler (CTDP) detector connected to the Barracuda electrometer (RTI Electronics, Mölndal, Sweden). The CTDP probe was designed specifically for CT dosimetry application.^(11)^ Collection and analysis of the data is performed with the software called “Ocean”, available from RTI. The signal collected is, in turn, sent to a laptop computer via USB cable (or wirelessly via Bluetooth). The laptop computer running the “Ocean” software captures the signal and displays the radiation waveform for analysis.

The second system is the Xi (Unfors Raysafe, Billdal, Sweden). The Xi detector is designed for application in conventional radiography and fluoroscopy dosimetry measurement.^9^, ^12^ As such, the Xi is backed with a lead support to shield any backscatter from being detected. Similarly, the signal obtained is sent to a laptop computer wirelessly via Bluetooth, and analysis of data is handled by the “Xi View” software.

Both RTI “Ocean” and Unfors “Xi View” software programs run under Microsoft Windows Operating System. The data obtained can be exported to Microsoft Excel spreadsheet program for further analysis, manipulation, and processing.

**Table 1:** Specifications of the detectors used in this study

*Detector*	*Software*	*Measurement Range*	*Time Measurement Accuracy*	*Temporal Resolution for 1200 ms Measurement*
CTDP	Ocean	<2000 s	±1%or±0.5ms	0.5 ms
Xi	Xi View	<1200 ms	±0.5%or±0.2ms	0.11 ms

### B. Measurement of rotation time

**Figure 1:**
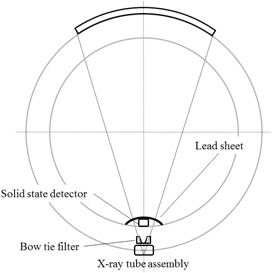
Experimental arrangement for the measurement of rotation time. The detector was positioned on the inner bottom of the gantry cover for gantry rotation time measurement under helical scan mode of operation. A lead sheet was placed on top of the detector to minimize the effect of unwanted primary radiation.

The detector (either CTDP or Xi) was positioned on the gantry cover, as shown in Fig. 1. Notice that to shield any stray radiation from reaching the detector, a sheet of lead (1 mm thick) is placed on top of the detector. Thus, the detector “sees” only the primary radiation as the X‐ray tube passes by the detector at the bottom of the gantry where the detector is located. Therefore, the time duration between two successive radiation signal peaks is the gantry rotation time. Since a pair of two successive peaks is used to determine the gantry rotation time, by necessity the helical scan must be employed, and the examination table may be in the imaging plane since its presence has no effect on the primary beam that enters the detector.

Upon completion of the data acquisition, the peak times were determined with the dedicated software. Subsequently, the rotation time is determined as the time between the two successive peaks.

## III. RESULTS

**Figure 2:**
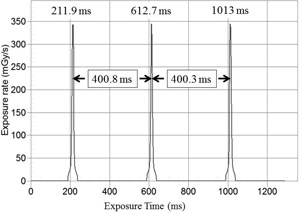
The detector output signals/peaks using RTI's CTDP as the detector. The CTDP was located at the off‐set position with a 400 ms nominal gantry rotation time. The three peaks are registered at 211.9, 612.7, and 1013 ms, respectively, The measured average gantry rotation time was 400.6 ms. (Fig. 2 is intended for illustration purposes only.)

Depicted in Fig. 2 is the dose‐rate profile from the CTDP probe as it was placed on the gantry cover. Using the cursor provided in the “Ocean” software, the peak times were determined. There are three peaks located at rotation times of 211.9, 612.7, and 1013 ms, respectively. Since any pair of two successive dose‐rate profiles (peaks) can be employed to determine the gantry rotation time, the average gantry rotation time is determined as 400.6 ms.

**Figure 3:**
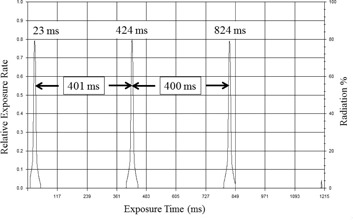
The detector output signals/peaks using Unfors’ Xi as the detector. The Xi detector was located at the off‐set position with a 400 ms gantry rotation time. The three peaks are registered at 23, 424, and 824 ms, respectively. The measured gantry rotation time was 400.5 ms. (Fig. 3 is intended for illustration purposes only).

Similarly, the dose‐rate profile obtained with the Xi detector, processed with “Xi View” software, is shown in Fig. 3. There are three peaks located at rotation time of 23, 424, and 824 ms, respectively. Therefore, the average gantry rotation time is determined as 400.5 ms.

## IV. DISCUSSION

CT technology has recently enabled high‐speed gantry rotation time of 300 ms or less,^(13)^ contributing to the high temporal resolution required for coronary CTA, cardiac function study, and pediatric CT examination. Thus, the gantry rotation time becomes an important scan parameter of any CT scanner. A shorter gantry rotation time also contributes to the possibility for increased length of coverage ($L_{c}$) which can be calculated as shown in Eq. (1):

(1) $$L_{c}=N\times W_{\text{Acq}}\times P\times T_{T}/T_{\text{RN}}$$

where $L_{c}$ is length of coverage (mm), *N* is number of data channel, $W_{\text{Acq}}$ is acquisition slice thickness (mm), *P* is pitch (IEC definition), $T_{T}$ is total scan time (sec), and $T_{RN}$ is gantry rotation time (sec/rotation), “nominal”. If inaccurate rotation time is set at installation in a modern CT system, the timing error may cause inaccurate pitch, image deformation along the z‐axis, and motion artifact in cardiac CT.

In this study, the physical parameter of interest is the rotation time, and the radiation dose (or dose rate) is not the primary parameter of interest. Therefore, the detector need not be placed at the isocenter for dosimetry. And the radiation detector may be affixed at an offset location to pick up the radiation signals as the X‐ray tube travels by the detector with multiple rotations. The rotation time can then be determined as the time between two successive radiation peaks, as shown in Figs. 2 and 3.

Under the respective software operation configuration, the temporal resolution of CTDP and Xi is 0.5 ms and 0.11 ms. Although Xi is 4.5 times faster than that of CTDP when gathering data for approximately 1200 ms, there was no large difference (>1.0 ms) in accuracy between the two detectors for measuring gantry rotation time. Therefore, these two solid‐state detectors can be applied to measure gantry rotation time without preference of one over the other.

It should be noted that this study had at least three limitations. First, at a nominal rotation time of 400 ms, to obtain three successive peaks would require a measurement of 1200 ms. The Xi detector does not allow for measurements in excess of 1200 ms. However, in the clinical environment, gantry rotation time can be longer than 1000 ms per rotation (e.g., cranial scanning^(14)^ and CT simulation for radiation treatment planning^(15)^). Therefore, Xi may not be adequate to measure the longer gantry rotation times. Furthermore, we did not verify how many peaks are needed for the precision of the measurement. Since the CTDP allows for measurements in excess of 1200 ms, many successive peaks can be obtained if necessary. In addition, any two consecutive peaks in a given run of a CT scan may be chosen for the measurement by adjusting the data collection initiation. A total measurement time of 1200 ms was also selected in order to maintain a better temporal resolution.

Second, the measurement was conducted only at 400 ms nominal rotation time. Since the rotation time controller of a modern CT scanner allows for selectable rotation time and is considered linear, it is necessary to evaluate the rotation time at other time settings for completeness. Although we had no access to state‐of‐the art CT systems with high‐speed gantry rotation time of 300 ms or less, at least, the temporal resolution of the both detectors (<0.5 ms) is adequate to measure it. Therefore, we believe these solid‐state detectors allow for high‐speed rotation time of 300 ms or less with a reasonable accuracy.

Third, all examinations were performed consecutively on the same day. As a result, the longterm stability of gantry rotation time has not been evaluated. Periodical QA for the stability of gantry rotation time may be necessary.

## V. CONCLUSIONS

Despite some of the shortcomings, a simple noninvasive method for the measurement of gantry rotation time in modern CT systems was introduced. It has been shown that measurements of CT scanner rotation time can be accomplished with a radiation probe designed for the CT application or with a conventional radiation probe designed for radiography and fluoroscopy applications. It is also noteworthy to point out that the measurement results are in good agreement between the two radiation detector systems. It is necessary to consider the dosimetry system's total length of time measurement permissible, since at least two radiation signal peaks would be required to determine the gantry rotation time. Depending on the detector system design, there will be a limitation on the maximum gantry rotation time that can be properly measured. Finally, clinical medical physicists should be aware of the accuracy and precision of gantry rotation time, and take this into consideration for QA where and when applicable.

## Supporting information

Supplementary Material

## References

[1] Zeman RK , Fox SH , Silverman PM , et al. Helical (spiral) CT of the abdomen. AJR Am J Roentgenol. 1993;160(4):719–25. 8456652 10.2214/ajr.160.4.8456652

[2] Kalender WA and Polacin A . Physical performance characteristics of spiral CT scanning. Med Phys. 1991;18(5):910–15. 1961153 10.1118/1.596607

[3] Jones TR , Kaplan RT , Lane B , Atlas SW , Rubin GD . Single‐versus multi‐detector row CT of the brain: quality assessment. Radiology. 2001;219(3):750–55. 11376264 10.1148/radiology.219.3.r01jn47750

[4] Mahesh M and Cody DD . Physics of cardiac imaging with multiple‐row detector CT. Radiographics. 2007;27(5):1495–509. 17848705 10.1148/rg.275075045

[5] Brodoefel H , Kramer U , Reimann A , et al. Dual‐source CT with improved temporal resolution in assessment of left ventricular function: a pilot study. AJR Am J Roentgenol. 2007;189(5):1064–70. 17954641 10.2214/AJR.07.2228

[6] Boone JM , Geraghty EM , Seibert JA , Wootton‐Gorges SL . Dose reduction in pediatric CT: a rational approach. Radiology. 2003;228(2):352–60. 12893897 10.1148/radiol.2282020471

[7] Lin PJ , Beck TJ , Borras C , et al. Specification and acceptance testing of computed tomography scanners. AAPM Report No. 39. Task Group 2 Diagnostic X‐Ray Imaging Committee, American Association of Physicists in Medicine. NY: American Institute of Physics; 1993 Retrieved May 1, 2013 from http://www.aapm.org/pubs/reports/rpt_39.pdf

[8] Mutic S , Palta JR , Butker EK , et al. Quality assurance for computed‐tomography simulators and the computed‐tomography‐simulation process. AAPM Report No. 83. Report of the AAPM Radiation Therapy Committee Task Group No. 66. Med Phys. 2003;30(10):2762–92. 14596315 10.1118/1.1609271

[9] Fukuda A , Miyati T , Matsubara K . Where should we measure the entrance air kerma rate during acceptance testing of the automatic dose control of a fluoroscopic system? Radiol Phys Technol. 2013 Retrieved July 1, 2013 from http://link.springer.com/article/10.1007%2Fs12194‐013‐0202‐9 10.1007/s12194-013-0202-9 23413079

[10] Lin PJ and Herrnsdorf L . Pseudohelical scan for the dose profile measurements of 160‐mm‐wide cone‐beam MDCT. AJR Am J Roentgenol. 2010;194(4):897–902. 20308488 10.2214/AJR.09.3048

[11] Palm A , Nilsson E , Herrnsdorf L . Absorbed dose and dose rate using the Varian OBI 1.3 and 1.4 CBCT system. J Appl Clin Med Phys. 2010;11(1):3085 Retrieved May 1, 2013 from http://www.jacmp.org/index.php/jacmp/article/viewArticle/3085 20160695 10.1120/jacmp.v11i1.3085 PMC5719770

[12] Vano E , Ubeda C , Leyton F , Miranda P . Radiation dose and image quality for paediatric interventional cardiology. Phys Med Biol. 2008;53(15):4049–62. 18612174 10.1088/0031-9155/53/15/003

[13] Fink C , Krissak R , Henzler T , et al. Radiation dose at coronary CT angiography: second‐generation dualsource CT versus single‐source 64‐MDCT and first‐generation dual‐source CT. AJR Am J Roentgenol. 2011;196(5):W550–W557. 21512044 10.2214/AJR.10.5153

[14] Alberico RA , Loud P , Pollina J , Greco W , Patel M , Klufas R . Thick‐section reformatting of thinly collimated helical CT for reduction of skull base‐related artifacts. AJR Am J Roentgenol. 2000;175(5):1361–66. 11044042 10.2214/ajr.175.5.1751361

[15] Nakamura M , Narita Y , Matsuo Y , et al. Geometrical differences in target volumes between slow CT and 4D CT imaging in stereotactic body radiotherapy for lung tumors in the upper and middle lobe. Med Phys. 2008;35(9):4142–48. 18841867 10.1118/1.2968096

